# Screening of a new candidate coxsackievirus B1 vaccine strain based on its biological characteristics

**DOI:** 10.3389/fmicb.2023.1172349

**Published:** 2023-07-12

**Authors:** Ming Zhang, Danhan Xu, Yuhan Liu, Xiaohui Wang, Lilan Xu, Na Gao, Changzeng Feng, Wei Guo, Shaohui Ma

**Affiliations:** ^1^Institute of Medical Biology, Chinese Academy of Medical Sciences, and Peking Union Medical College, Kunming, China; ^2^Yunnan Key Laboratory of Vaccine Research Development on Severe Infectious Disease, Kunming, China

**Keywords:** coxsackievirus B1, Vero cell, murine model, inactivated vaccine, immunogenicity

## Abstract

Coxsackievirus B1 (CVB1) is one of the significant pathogens causing viral myocarditis, hand, foot, and mouth disease (HFMD), and aseptic meningitis, and it has been associated with type 1 diabetes (T1DM). No effective antiviral drugs against CVB1 infection or preventive vaccines are available. Due to the success of two inactivated vaccines against enterovirus 71 and poliovirus, an inactivated Vero cell-based CVB1 vaccine could be developed. In this study, we isolated a high-growth CVB1 virus strain KM7 in Vero cells and developed a Vero-adapted vaccine candidate strain KM7-X29 via three rounds of plaque purification and serial passages. The KM7-X29 strain was grouped into the GII sub-genotype, which belonged to the Chinese epidemic strain and grew to a titer of more than 10^7^ CCID50/ml in Vero cells. The inactivated CVB1 vaccine produced by the KM7-X29 strain induced an effective neutralizing antibody response in BALB/c mice, and maternal antibodies were able to provide a 100% protective effect against lethal challenges with a CVB1 strain in suckling BALB/c mice. Thus, the KM7-X29 strain might be used as a new candidate coxsackievirus B1 vaccine strain. The neonatal murine model of CVB1 infection will contribute to the development of the CVB1 vaccine.

## 1. Introduction

Coxsackievirus B1 (CVB1) is one of the major members of the Coxsackievirus Bs (CVBs) (CVB1 to CVB6), belonging to the genus *Enterovirus* of the Picornaviridae family. CVB1 is a non-enveloped, positive-stranded RNA virus. The genome is ~7,400 nucleotides in length, with a single open reading frame (ORF) encoding a 2,182-amino-acid polyprotein. The ORF is covalently connected at the 5′-end and has a poly(A) tail at the 3′-end (Iizuka et al., 1987). The polyprotein can be cleaved into three intermediate products (P1, P2, and P3), and these products are further processed to produce four structural proteins (VP4, VP3, VP2, and VP1) and seven non-structural proteins (2A, 2B, 2C, 3A, 3B, 3C, and 3D) (Nasri et al., 2007). The VP1 protein shows the highest sequence variation between enteroviruses (EVs) but also contains a conserved immunodominant epitope (Oberste et al., 1999). Since the CVB1 prototype strain Conn-5 was isolated in 1948 (Quinn et al., 2014), CVB1 isolates have evolved into eight clusters based on the VP1 gene (Chu et al., 2015). The Chinese CVB1 isolates belong to cluster GII (Chu et al., 2015).

CVB1 infections are usually subclinical. In China, since hand, foot, and mouth disease (HFMD) was classified as a Class C statutory infectious disease in May 2008, CVB1 is frequently detected in HFMD outbreaks or sporadically (Guan et al., 2015; Chen et al., 2019; Ji et al., 2019). Except for HFMD, CVB1 also causes life-threatening infections, such as encephalitis (Jain et al., 2014), myocarditis (Verma et al., 2009), aseptic meningitis (Lee et al., 2022), and necrotizing hepatitis (Wong et al., 1989; Wang et al., 1998), and CVB1 can even cause death (Wikswo et al., 2009). CVB1 has also been confirmed to be associated with type 1 diabetes (T1DM) (Oikarinen et al., 2014; Sioofy-Khojine et al., 2018). In 2007, CVB1 was the most frequently reported EV serotype in the United States (accounting for 25% of all reported cases of EV infection) (Centers for Disease Control Prevention (CDC), 2008). In 2009, there was a significant increase in cases of severe CVB1 infection in South Korea (Kim et al., 2013). A recent study has shown that CVB1 has become one of the main pathogens causing HFMD in China (Kim et al., 2013). CVB1 has also been associated with severe HFMD (Kim et al., 2013; Ji et al., 2019; Guo et al., 2022). In 2017, T1DM accounted for ~2% of all estimated cases of diabetes, ranging from <1% in some Pacific countries to >15% of the northern European populations (Green et al., 2021). Thus, CVB1 has emerged as a critical EV serotype that poses a substantial public health threat. Although the triple combination of pleconaril, guanidino-HCl, and oxoglaucine recently showed considerable protection in neonatal mice infected with a neurogenic CVB1 strain by sequential alternate administration, it only reduced mortality and increased mean survival time (Stoyanova et al., 2015a,b). However, no effective antiviral agents against CVB1 infection or preventive vaccines are available. At present, to protect against EV infections, only two approved vaccines are available against polio (Kersten et al., 1999) and enterovirus 71 (EV-A71) (Reed and Cardosa, 2016). EV vaccines contain either attenuated live viruses (oral polio vaccine) or inactivated whole viruses (inactivated EV-A71 vaccine and polio vaccine). The African green monkey kidney cells (Vero cells) are widely used in the production of inactivated vaccines against EV-A71, poliovirus, and COVID-19, and they pose a low health risk, suggesting that they are suitable for the production of a human-inactivated CVB1 vaccine. Although an inactivated CVB1 vaccine was previously developed based on Vero cell (Hankaniemi et al., 2019a), the CVB1 vaccine strain Nm (EU147493) was of the same genotype as the CVB1 prototype, and the whole-genome nucleotide and amino acid identities between the two strains were 99.7 and 99.4%, respectively (Stone et al., 2020). However, the strain Nm showed 79.0–80.8% nucleotide and 95.0–95.6% amino acid identity with other prevalent Chinese strains. Moreover, the previous study found significant differences in cross-neutralization between the prevalent and the prototype strains (Liu P. et al., 2021). The inactivated CVB1 vaccine may not be very effective against the epidemic CVB1 infection in China. Therefore, we hope to develop a new inactivated CVB1 vaccine by screening the currently prevalent strains of CVB1 in China. In this study, we isolated 20 CVB1 strains from 20 stool samples of patients with HFMD on Vero cells and screened a strain named KM7. After three rounds of plaque purification in Vero cells, the KM7-X29 clone was obtained because its complete genome sequence is identical to the original strain KM7. Then, 15 serial passages of KM7-X29 were performed, and the genetic stability of the virus was assessed. Because the complete genome sequence of KM7-X29 P10 is identical to KM7-X29 P15, the growth and virulence of the virus were assessed. The manufacturing process for producing a new CVB1 vaccine based on the KM7-X29 P10 strain was established, including virus harvesting time, concentration, purification, and virus inactivation methods. The new inactivated vaccine candidate formulated with aluminum adjuvant elicited the production of a neutralizing antibody (NAb) and protected against CVB1 infection in mice. These results demonstrated that the KM7-X29 strain might be a new suitable vaccine strain.

## 2. Materials and methods

### 2.1. Ethics statement

BALB/c mice were obtained from the Laboratory Animal Center, Institute of Medical Biology, Chinese Academy of Medical Sciences [SYXK(Dian)K2019-0002]. All animal experiments were approved by the Animal Ethics Committee of the Institute of Medical Biology, Chinese Academy of Medical Sciences (DWLL202108001). Experiments were performed following the animal welfare guidelines of the People's Republic of China.

### 2.2. Cells and viruses

**Figure 1:**
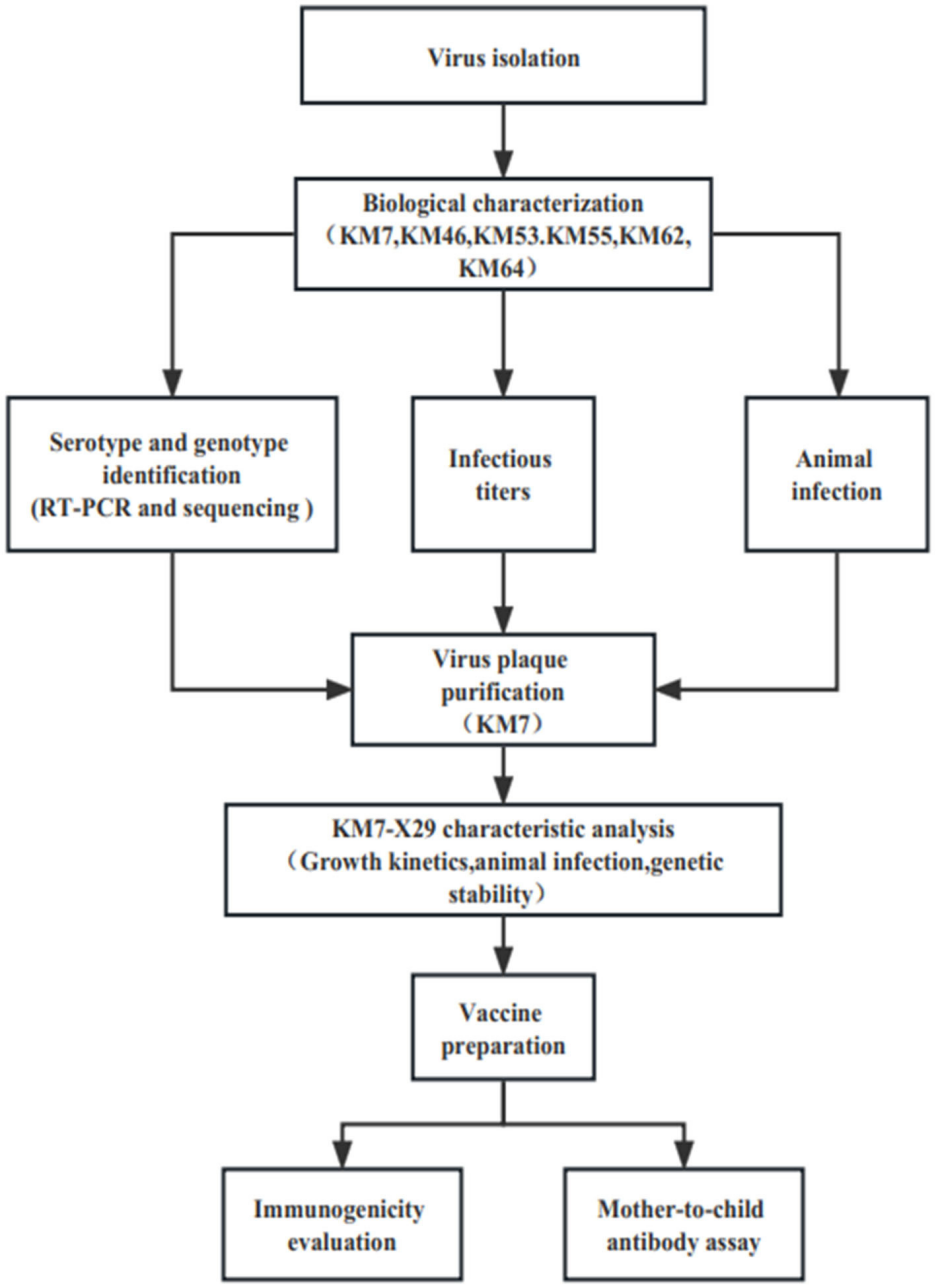
The flowchart of the study.

Twenty CVB1 strains were isolated from the stools of HFMD patients from 2010 to 2019 during HFMD surveillance in Yunnan, China, according to the standard procedures recommended by the World Health Organization (WHO). In brief, 20% suspensions of stools were made in the phosphate-buffered saline (PBS) and centrifuged at 3,000 *g* for 30 min at 4°C. Then, the suspension treated with a 0.22-μm filter was inoculated with the human rhabdomyosarcoma (RD), green monkey kidney (Vero), and human embryonic lung diploid fibroblast (KMB17) cells. Then, the three cells were grown into confluent monolayers and were incubated in the presence of 5% CO_2_ at 37°C. The cytopathic effect (CPE) was observed under a microscope daily, and each sample was passed blindly for three generations. The positive samples were stored at −80°C. Viruses were propagated in the three cells using a minimum essential medium (MEM) solution containing 10% fetal bovine serum (MINHAI BIO, China), 2 mM l-glutamine, and 100 IU/ml penicillin and streptomycin, respectively. EV-A71 strain 66V3, Coxsackievirus A16 (CVA16) strain 127V3, Coxsackievirus A6 (CVA6) strain KYA1205, Coxsackievirus A6 (CVA10)strain V6-19, CVB1 prototype strain Conn-5, CVB2 strain 31V3, CVB3 strain 20V3, CVB4 strain 136V3, and CVB5 strain 03V3 were preserved at the Institute of Medical Biology, Chinese Academy of Medical Sciences, and Peking Union Medical College (Kunming, China). The flowchart of the study is presented in Figure 1.

### 2.3. RT-PCR, sequencing, and typing

The VP1 sequences and complete genomes were amplified and typed as described previously (Liu et al., 2015). In brief, the viral RNA from the infected cell supernatants was extracted using a QIAamp Viral RNA Mini Kit (Qiagen, USA). The partial VP1 sequences and complete genomes were amplified using the PrimeScript One Step RT-PCR Kit version 2 (Takara, Dalian, China) and sequenced using an ABI 3130 Genetic Analyzer (Applied Biosystems, USA). Primers for amplification and sequencing are shown in Supplementary Table 1. EV types were classified using the Enterovirus Genotyping Tool (https://www.rivm.nl/mpf/typingtool/enterovirus/).

### 2.4. Phylogenetic analysis and sequences alignments

The Molecular Evolutionary Genetic Analysis (MEGA) software version 7.0 (the neighbor-joining method) was used to construct the phylogenetic analysis of 60 CVB1 strains (including 40 strains from the GenBank database and 20 strains isolated in this study) based on the complete VP1 sequence (876nt). The alignments of these sequences were analyzed using Geneious 9.0.2 software.

### 2.5. Infectious titers of the virus

The infectious titers of the virus were determined by a microtissue culture technique (Liu H. et al., 2021). In brief, serial 10-fold dilutions of the samples were inoculated into Vero cells in 96-well plates and cultured at 37°C in the presence of 5% CO_2_ for 7 days. Then, the 50% cell culture infectious doses (CCID50) were quantified using the Reed–Muench method.

### 2.6. Animal infection

In total, 3-day-old BALB/c mice were intracerebrally (i.c.) injected with CVB1 KM7, KM46, KM53, KM55, KM62, and KM64 strains at a dose of 10^4^ CCID50/mouse_._ Mice in the control group were i.c. injected with uninfected cell supernatant (30 μl/mouse) and housed separately from infected mice. The dose and age of the attacked mice were screened (data not shown). Mice were monitored for 6 days. The scores of clinical symptoms were evaluated according to the following criteria: 0, healthy; 1, lethargy and inactivity; 2, emaciation; 3, limb weakness, hypotrichosis, hunchback, and/or inability; and 4, moribundity or dead (Wang et al., 2018).

### 2.7. Virus plaque purification

To obtain the pure CVB1 strain, the KM7 strain was purified in Vero cells by the three cycles of plaque assay. In brief, 10-fold gradient dilution (10^−2^ to 10^−6^) was added to Vero cells and incubated at 37°C and 5% CO_2_ for 1 h. Then, after the supernatant was removed, 2 ml of MEM containing 3% methylcellulose was added to cells and incubated at 37°C and 5% CO_2_. The plaques were observed and selected under a microscope, and the selected plaques were inoculated in 96-well or 24-well plates containing Vero cells and observed CPE under a microscope daily. When the viral RNA from the positive samples was identified as CVB1, as discussed above, the next round of plaque purification was carried out. Each virus strain was purified three times using the same method.

### 2.8. Genetic stability of KM7-X29 strain

To provide a reference for the establishment of a seed bank for inactivated CVB1 vaccine, the clone strain KM7-X29 was successively passaged for 15 to obtain its genetic stability and the large amount of viral stock in Vero cells. The whole genomes of P1, P5, P10, and P15 were amplified and sequenced as above. The infectious titers of P1, P5, P10, and P15 were quantified using the Reed–Muench method. In the continuous cultivation, the virus obtained by inoculating Vero cells with KM7-X29 was labeled the P2 generation.

### 2.9. Growth kinetics of CVB1 KM7-29 strain on Vero cells

Vero cells (90–95% confluent monolayer) were infected by clone KM7-X29 P10 at a multiplication of infection (MOI) of 0.001, 0.01, 0.1, 1, or 10. After adsorption for 2 h at 37°C, the viral fluid was removed. Then, the cells were washed with PBS three times, 1 ml of MEM was added, and cells were incubated at 35°C. At 0, 1, 2, 4, 8, 16, and 24 h, cells with MOIs of 1 and 10 were harvested, and at 1 to 9 days, cells with MOIs of 0.001, 0.01, and 0.1 were harvested. All the flasks were freeze-thawed three times and stored at −80°C. Three replicates were set up at each time point. The infectious titers of the virus were determined using the Reed–Muench method.

### 2.10. Histopathology and immunohistochemistry analyses

Histopathology and immunohistochemistry (IHC) analyses were carried out as previously reported (Liu H. et al., 2021). In brief, 3-day-old BALB/c mice were inoculated with KM7-X29 P10 strain (10^4^ CCID50/mouse), and mice with a clinical score of 4 were anesthetized with ether and sacrificed. The tissues were separated, fixed in 4% formalin, dehydrated, permeabilized, and embedded in paraffin. The sections were sliced, and after staining with hematoxylin and eosin (HE), the histopathology was observed under a microscope. For IHC analysis, the tissue sections were dewaxed, dehydrated, and boiled for 15 min. Polyclonal rabbit anti-CVB1 antibody (1:1000 dilution) was applied at 4°C overnight. After washing, samples were incubated with goat anti-rabbit secondary antibody for 50 min at room temperature. Signals were visualized using a histochemical DAB color development kit (Servicebio, Wuhan, China). After hematoxylin staining and desiccation, sealed slides were detected under a microscope.

### 2.11. CVB1 viral loads in different organs

The viral loads in different tissues of KM7-X29 P10**-**infected mice were determined as previously reported (Liu H. et al., 2021). In brief, at 6, 12, 24, and 48 h after the challenge, the pancreas, heart, liver, spleen, lung, kidney, small intestine, forelimb muscles, hindlimb muscles, and brain tissues were separated, weighed, and ground employing a high-speed grinder (KZ-II, Servicebio, Wuhan, China). The total RNA of individual tissues was extracted with the TRIzol reagent (Invitrogen, Carlsbad, CA, United States). Then, the RNA load was determined using the One Step PrimeScript™ RT-PCR Kit for Perfect Real-Time PCR (Takara, Dalian, China). Primers CVB1-qP-F (5′-GACTGGGCATACCTCCCAAGT-3′) and CVB1-qP-R (5′-ATTCGGACCGTGAATGGTAGTTT-3′) as well as the CVB1 probe (6-FAM-CCTAGYGACACCATGCAAACAAGACAYG-BHQ1) were used. In addition, a recombinant plasmid containing the VP1 gene of CVB1 was constructed and linearized. The VP1 RNA of CVB1 obtained by *in vitro* transcription was used to assess the number of CVB1 copies.

### 2.12. Preparation of inactivated whole-virus CVB1 vaccine

CVB1 KM7-X29 P10 strain was inoculated in Vero cells at an MOI of 0.01. When 90–95% CPE was observed, the culture was harvested by three freeze–thaw cycles, and the samples were filtered through a 0.45-μm filter to remove the cellular debris. The supernatant was mixed with formaldehyde solution (Sigma–Aldrich, MO, United States) at a volume ratio of 4000:1, incubated at 37°C for 7 days for virus inactivation, and filtered using a 0.22-μm filter (Millipore, MA, United States). No live virus was detected during three consecutive blind passages on Vero cells. The inactivated supernatants were concentrated by ultrafiltration using a 100 kDa PES membrane (Millipore, MA, USA) and purified using a Capto Core 400 gel column to prepare the inactivated CVB1 vaccine. The vaccine was formulated in M199 medium and aluminum adjuvant.

### 2.13. Active immunization assay of CVB1 inactivated vaccine

Three groups of 4-week-old female BALB/c mice (*n* = 6 per group) were subcutaneously injected with 1, 5, 10, or 20 μg of the inactivated CVB1 vaccine in 100 μl on days 0 and 14, respectively. Serum samples were collected before days 0, 14, and 35. The two negative controls were injected with the same volume of adjuvant and M199, respectively. All mice were monitored daily for clinical symptoms until 21 days post-immunization (dpi). The inactivated CVB1 vaccines were formulated with aluminum adjuvant from M199. The NAb titer was determined as previously reported (Liu H. et al., 2021).

### 2.14. Neutralizing antibody assay

The NAb titer was determined as previously reported (Qian et al., 2021). In brief, the CVB1 antisera collected from immunized and control mice were heat-inactivated at 56°C for 30 min and 2-fold serially diluted in MEM; subsequently, 50 μl of the diluted antiserum samples was mixed with 50 μl of 100 CCID50 CVB1 in 96-well plates in three replicates. After incubation at 37°C for 2 h, 10^4^ Vero cells/well were added to the 96-well plates. Vero cells and antiserum were used as the control. At the same time, virus back-titration was carried out as follows: 50 μl CVB1 virus was 10-fold diluted in four replicates for each dilution and mixed using 50 μl MEM without fetal bovine serum (FBS). Then, 1 × 10^4^ Vero cells in 100 μl were added to the 96-well plates. After 7 days, the infectious titers of the virus were 32–320 CCID50/50 μl. The NAb titers were calculated as the highest dilution with 50% inhibition of CPE.

### 2.15. Maternal antibody protection experiment

In total, 8-week-old female BALB/c mice were subcutaneously injected with 20 μg of the inactivated CVB1 vaccine or the same volume of M199 medium. When the female mice were pregnant, boost immunization was carried out. After delivery, 3-day-old BALB/c mice were i.c. injected with 10^6^ CCID50 of the homologous CVB1 KM7-X29 strain, and the body weight and clinical scores were observed for 7 days.

### 2.16. Statistical analysis

All data were statistically analyzed using GraphPad Prism 9.0.0 (San Diego, California, United States). Comparisons were conducted using the one-way ANOVA, log-rank test, and *t*-test, and *p* < 0.05 was considered statistically significant.

### 2.17. Accession numbers

The genome and VP1 gene sequences of the clinical isolates characterized in the present study were R461/YN/CHN/2010, R42/YN/CHN/2014, R43/YN/CHN/2014, K148/YN/CHN/2014, V1444/YN/CHN/2014, R157/YN/CHN/2015, K1512/YN/CHN/ 2015, R1516/YN/CHN/2015, R1539/YN/CHN/2015, R1552/YN/ CHN/2015, R1570/YN/CHN/2015, V1573/YN/CHN/2015, V1578/ YN/CHN/2015, R1587/YN/CHN/2015, KM7/YN/CHN/2019, KM46/YN/CHN/2019, KM53/YN/CHN/2019, KM55/YN/CHN/ 2019, KM62/YN/CHN/2019, and KM64/YN/CHN/2019, which have been deposited in GenBank under accession numbers OP917832–OP917851. The complete genome sequences of KM7-X29 generations 1, 5, 10, and 15 have been uploaded to GenBank with gene accession numbers OQ259526-259529.

## 3. Results

### 3.1. Primary characterization of the virus isolates

**Figure 2:**
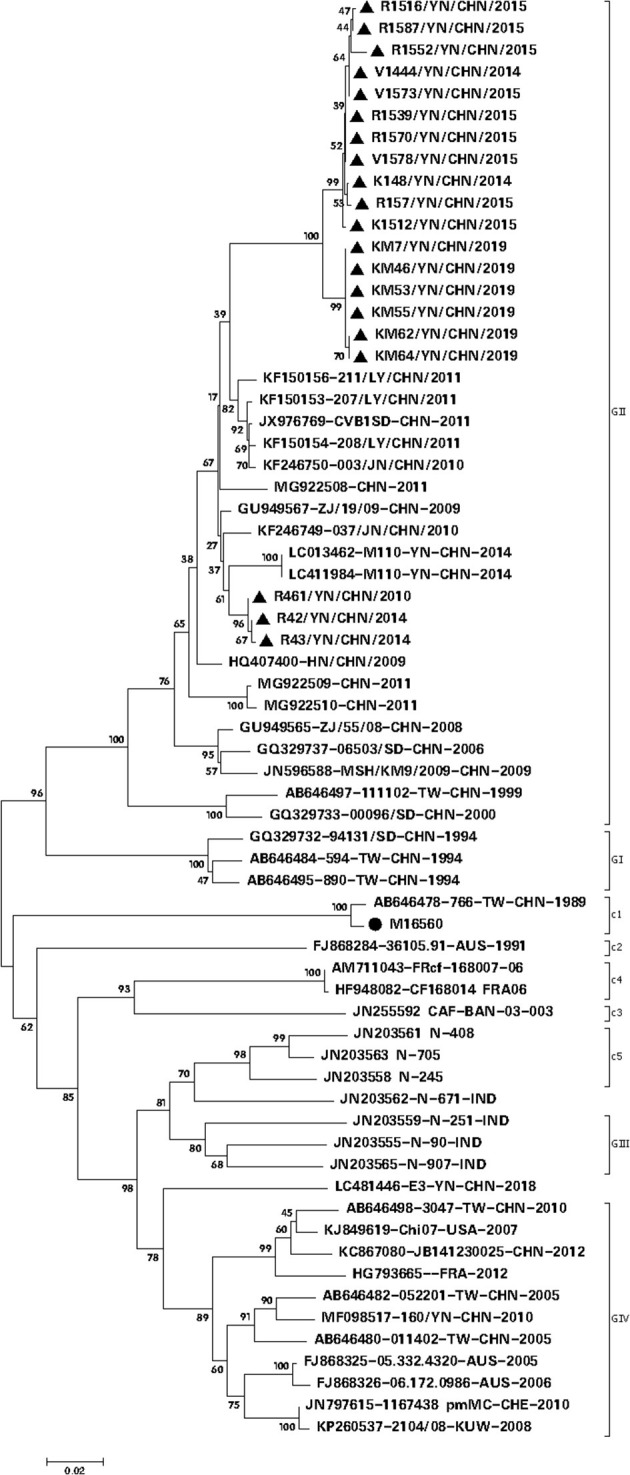
Phylogenetic analysis based on the complete VP1 gene sequence (834 nt) of CVB1 (20 isolates from this study and 40 representative CVB1 strains from GenBank) (parameter test of phylogeny: bootstrap method; No. of bootstrap replications: 1,000; mode: Kimura 2-parameter model), showing a bootstrap value of >70%. The symbol • represents the CVB1 prototype strain, while the symbol ▴ indicates the CVB1 strain isolated in this study.

Twenty CVB1 strains were isolated from RD, KMB17, and Vero cells (Supplementary Table 2). Two CVB1 strains were recovered from KMB17 cells, nine were from Vero cells, and nine were from RD cells. After adaptive culture in Vero cells, only six Vero cell-adapted strains (KM7, KM46, KM53, KM55, KM62, and KM64) could produce an evident cytopathic effect (CPE) within 3–5 days, and their infection titers were from 10^6.0^ CCID50/ml to 10^6.875^ CCID50/ml, respectively. Among them, KM7 Vero cell-adapted strain had the highest virus titer (10^6.875^ CCID50/ml) and showed the most typical CPE, which indicated that the KM7 strain had the most adaptability in Vero cells. Phylogenetic analysis based on VP1 revealed that the 20 CVB1 isolates grouped with epidemic strains from China and belonged to the GII genotype (Figure 2). Nucleotide and amino acid sequence homologies of the whole VP1 sequences among them were 94.0–100% and 97.8–100%, respectively. Furthermore, they shared 78.3–78.5% nucleotide identity and 85.7–86.4% amino acid identity with the whole VP1 sequence of the CVB1 prototype Conn-5 strain (M16560).

### 3.2. The virulence of six CVB1 Vero cell-adapted strains in BALB/c mice

**Figure 3:**
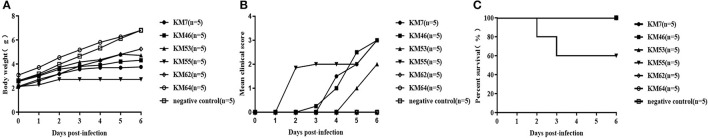
Virulence of six CVB1 isolates in BALB/c mice. Three-day-old (upper panels, 10^4^ CCID50/mouse) BALB/c mice were i.c. inoculated with KM7, KM46, KM53, KM55, KM62, or KM64. Control animals were inoculated with an equal volume of uninfected cell supernatant instead of the virus. The body weight **(A)**, mean clinical score **(B)**, and percent survival **(C)** in each group of neonatal mice were measured.

Six CVB1 Vero cell-adapted strains (KM7, KM46, KM53, KM55, KM62, and KM64) with highly infectious titers (>10^6.0^ CCID50/ml, Supplementary Figure 1) were screened to evaluate their virulence in the 3-day-old BALB/c mice by i.c. injection at 10^4^ CCID50/mouse. Clinical symptoms and the survival rate of these mice were monitored for 7 days (Figure 3). The mice infected with KM55 exhibited weakness, emaciation, and no weight gain; two died at 2 and 3 dpi, respectively. Mice infected with KM7, KM46, and KM53 gradually developed clinical symptoms, including emaciation and limb weakness, within 3–7 dpi but did not die. In mice infected with KM62 and KM64, no clinical symptoms were observed. In addition, no clinical symptoms were observed in the MEM-inoculated negative control group. These results showed that KM55 was the most virulent in BALB/c mice; KM7, KM46, and KM53 showed moderate virulence; and KM62 and KM64 were the least virulent.

### 3.3. The plaque purification of KM7 strain

According to the above results, the KM7 strain was screened and purified by three rounds of plaque. After three rounds of plaque purification, 30 clone strains were obtained. However, by comparing the VP1 gene and complete genome sequences of the 30 clone strains, a clone strain (KM7-X29) was selected for further study because the complete genome sequence of clone KM7-X29 is identical to the original strain KM7.

### 3.4. Genetic stability of KM7-X29 strain

No nucleotide mutations were observed in the whole-genome sequence of the clone KM7-X29 compared with the original strain KM7 by sequencing and analysis of the whole genomes of the P1 generation. However, by comparing the complete genome sequences at the P1, P5, P10, and P15 generations, three nucleotide mutations were observed in the VP1 gene (2692 A>G, 2698 G>A, and 2906 A>C), resulting in three amino acid substitutions (652 N>D for P5, 654 E>K for P5, P10, and P15, and 723 K>T for P10 and P15) in the VP1 protein (Table 1). Nevertheless, the nucleotide and amino acid sequence homologies among the whole genome of those offspring strains were 99.92–100 and 99.78–100%, respectively. In addition, except for the P1 generation, its infectivity titer was 10^5.75^ CCID50/ml, and the infectivity titers of P5, P10, and P15 were above 10 ^7.0^ CCID50/ml (Supplementary Figure 2), which suggested that the clone strain KM7-X29 has good genetic stability in Vero cells.

**Table 1:** Comparison of the whole-genome sequences of the different KM7-X29 offspring viruses.

**Virus**	**Site**		**Bases or amino acids of different generations**			
	**Nucleic acid**	**Amino acid**	**P1**	**P5**	**P10**	**P15**
KM7-X29	2692	/	A	G	A	A
	2698	/	G	A	A	A
	2906	/	A	A	C	C
	/	652	N	D	N	N
	/	654	E	K	K	K
	/	723	K	K	T	T

Nucleotide positions were referenced to the whole genome sequence of KM7-X29 P1 strain. The full names corresponding to each amino acid abbreviation are: N (asparagine), D (aspartic acid), E (glutamic acid), K (lysine), and T (threonine).

### 3.5. Growth characteristics of KM7-X29 P10 strain

**Figure 4:**
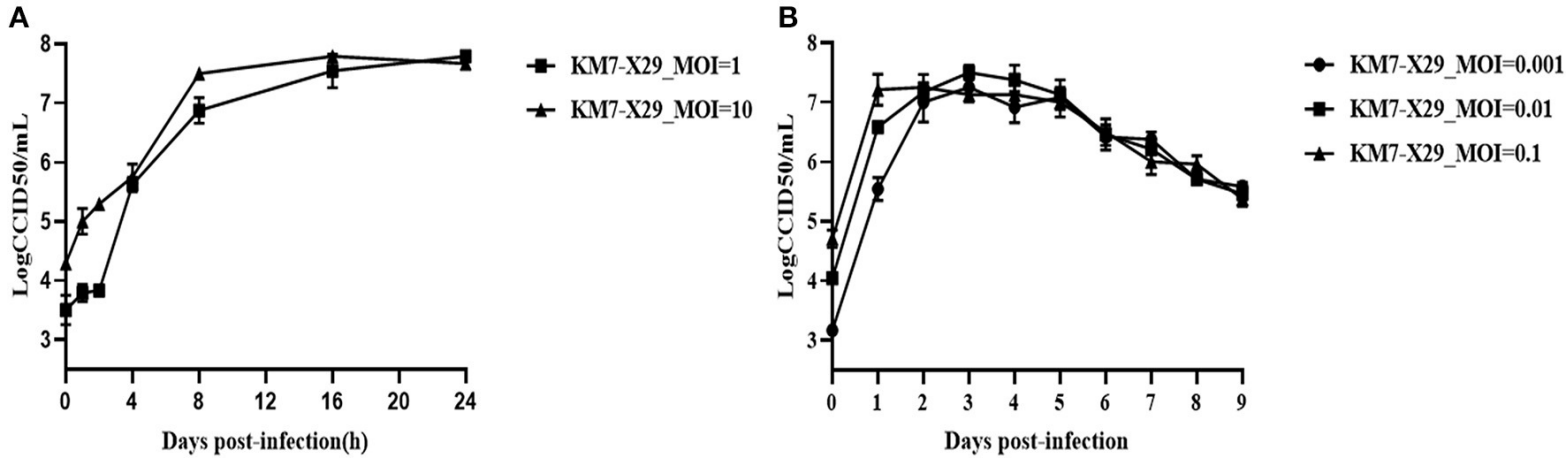
Growth curve of KM7-X29 in Vero cells. **(A)** The viral titers of KM7-X29 were determined by a CCID50 assay at 0, 1, 2, 4, 8, 16, and 24 h post-infection at MOIs of 1 and 10. **(B)** The viral titers of KM7-X29 were determined using a CCID50 assay on days 1, 2, 3, 4, 5, 6, 7, 8, and 9 post-infection at MOIs of 0.001, 0.01, and 0.1. Data are shown as mean ± SEM.

To study the growth characteristics of the working virus seed stock KM7-X29 P10, Vero cells were infected using various MOIs (0.001, 0.01, 0.1, 1, and 10, respectively). At an MOI of 1, 10^7.79^ CCID50/ml was detected at 24 h post-infection. At an MOI of 10, the virus titers peaked (10^7.5^ CCID50/ml) at 16 h post-infection and then declined (Figure 4A). At an MOI of 0.01, CVB1 KM7-X29 P10 showed the best growth kinetics, with the viral titer peaking at day 3 (10^7.5^ CCID50/ml). However, under high MOI infection (MOI = 0.1), viral titers peaked at 48 h post-infection (10^7.25^ CCID50/ml). At a low MOI of 0.001, viral titers peaked at 72 h post-infection (10^7.25^ CCID50/ml). On the fifth day after infection, the viral titer gradually decreased from 10^7.0^ to 10^5.0^ CCID50/ml for the three MOIs (Figure 4B). These results suggest that an MOI of 0.01 would help simplify downstream processing for the production of inactivated CVB1 vaccine.

### 3.6. Histopathological and immunohistochemical analyses of KM7-X29 P10 strain

**Figure 5:**
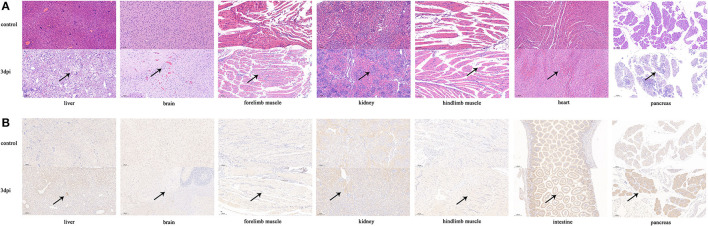
Histopathology and IHC analyses of tissues from KM7-X29 P10-infected neonatal BALB/c mice (×100). Histopathology **(A)** and IHC **(B)** of various tissues from CVB1-infected neonatal BALB/c mice were analyzed. Three-day-old BALB/c mice were i.c. inoculated with 10^4^ CCID50 of KM7-X29 P10. Sections from the heart, liver, pancreas, kidney, brain, and forelimb and hindlimb muscle tissues were stained with hematoxylin to detect pathological changes. Viral proteins were detected in IHC assays with an anti-CVB1 whole-virus polyclonal antibody as the primary antibody. Black arrowheads indicate representative inflammatory cell infiltration and expression.

Histopathological analyses showed that the pancreas, brain, and liver of infected mice exhibited severe cellular necrosis with inflammatory infiltration; punctate or focal necrosis was observed in the heart and limb muscle, and large numbers of renal tubular epithelial cells with cytoplasmic laxity were observed (Figure 5A). No histopathological change was observed in other detected tissues (the spleen, lung, and intestine) of the infected mice. IHC analyses showed that CVB1 antigens were also present in the pancreas, brain, liver, forelimb muscle, kidney, and intestine (Figure 5B). However, no viral antigen was observed in the other tissues of the infected mice, such as the heart, spleen, and lung (data not shown). No histopathological change was observed, and no antigen was detected in the control groups.

### 3.7. Viral loads in different organs of KM7-X29 P10-infected mice

**Figure 6:**
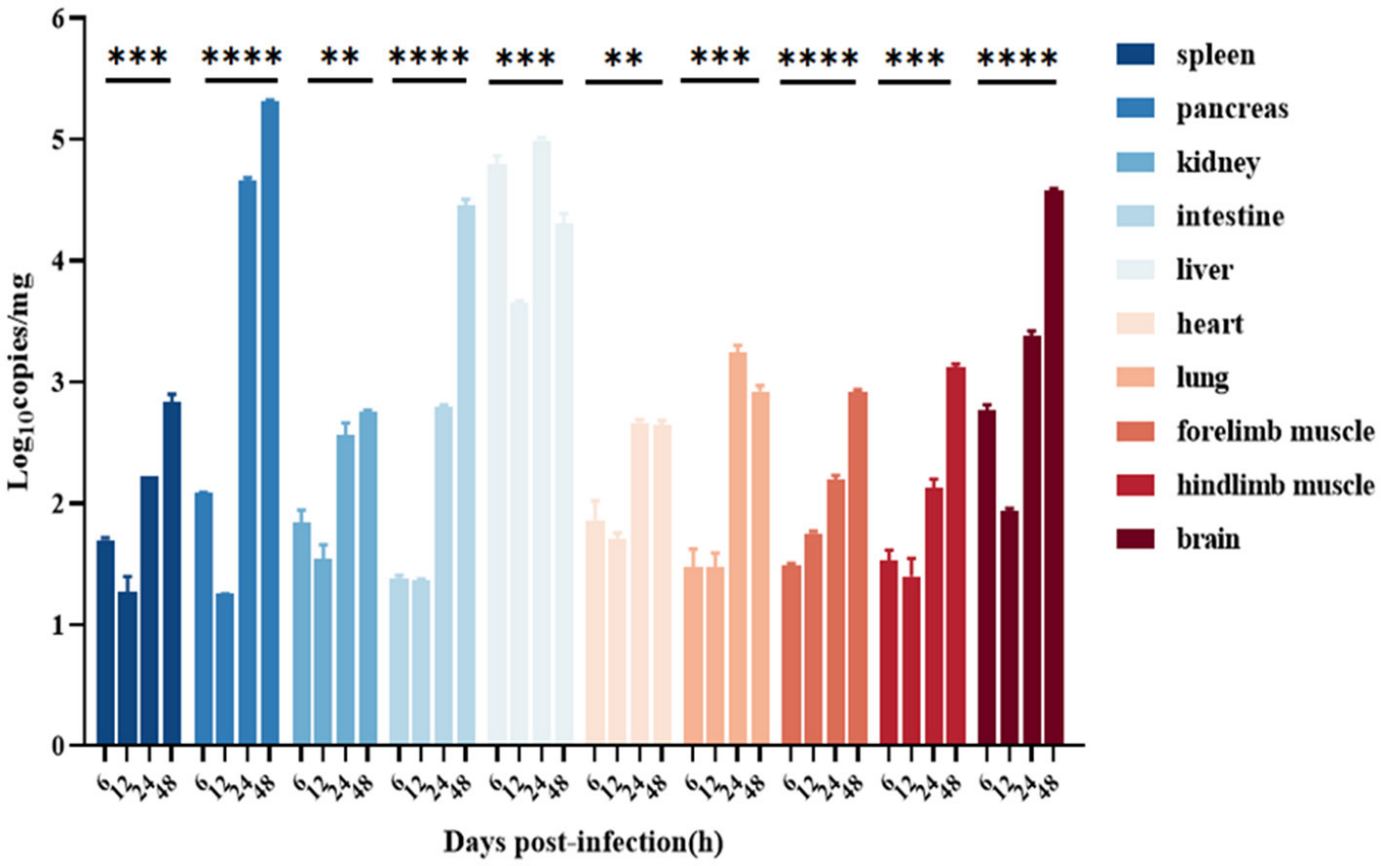
Distribution in various organs of the CVB1 strain KM7-X29 P10-infected 3-day-old BALB/c mice. Viral loads were assessed using qRT-PCR and compared with standard curves obtained from 10-fold serial dilutions of CVB1 transcript. Data are presented as the mean ± SEM (*n* = 3 mice per group) and analyzed using the one-way ANOVA (**, ***, and **** denote *p* < 0.01, *p* < 0.001, and *p* < 0.0001, respectively).

The viral loads of the liver were the highest at 6–48 h after infection; the CVB1 copy number in the pancreas was significantly increased after 24 h, reaching 10^5.4^ copies/mg at 48 h; and the CVB1 copy numbers in the brain and the intestine were significantly increased at 48 h, reaching 10^4.3^ copies/mg, which was consistent with the HE staining and IHC results. In addition, at 8 h after infection, among all tissues, the viral load in the front and hind limbs was the highest (Figure 6).

### 3.8. Immunogenicity evaluation of an inactivated CVB1 vaccine

**Figure 7:**
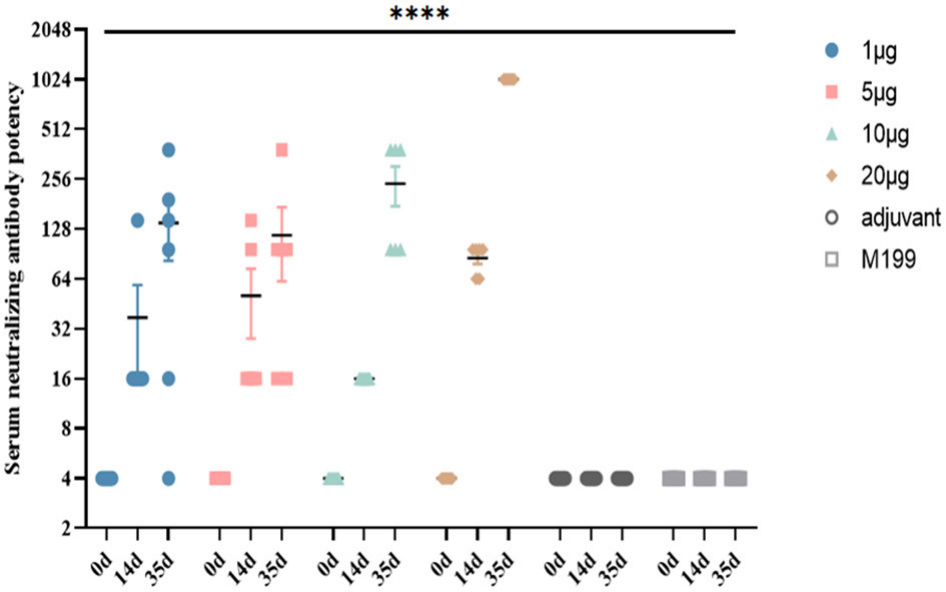
Dynamic profiles of neutralizing antibody responses induced by different doses of inactivated CVB1 vaccine in BALB/c mice at different immunization intervals. In total, 4-week-old female BALB/c mice (6 mice per group) were immunized with 1, 5, 10, and 20 μg of inactivated CVB1 vaccine, and the mice received two booster immunizations within 2 weeks. The blood samples were collected on days 0, 14, and 35. M199 containing aluminum hydroxide and M199 medium were used as the control. Data are expressed as mean ± SEM and analyzed using the one-way ANOVA (****indicates *p* < 0.0001).

BALB/c mice were vaccinated with the inactivated CVB1 vaccine, prepared using KM7-29X P10 strain, which could be induced to produce highly potent NAbs. The NAb levels in the 1, 5, and 10 μg groups were significantly increased 21 days after the second immunization, reaching 1:280 to 1:507. In the 20 μg group, the NAb level reached 1:1024 21 days after the second immunization, which was higher than the antibody level induced by two low-dose immunizations (Figure 7). No abnormality was observed in the vaccinated mice, indicating that the vaccine was safe.

To test whether anti-CVB1 antiserum can neutralize different clinical CVB1 isolates (KM46, KM53, KM55, KM62, and KM64), EV-A71, CVA16 strain, CVA6 strain, CVA10, CVB1 prototype strain Conn-5, CVB2, CVB3, CVB4, and CVB5 infection, 1024-fold dilution of the antiserum was incubated with these EVs following inoculation in Vero cells, respectively. The results of the cross-neutralization assays revealed that the clinical CVB1 isolates could significantly be neutralized by antiserum against the inactivated CVB1 vaccine, and the neutralization titers of antiserum induced in the 20 μg groups against these EVs were all 1:1,024. However, the CVB1 antiserum did not neutralize EV-A71 (66V3), CVA16 (127V3), CVA6 (KYA1205), CVA10 (V6-19), CVB1 (Conn-5), CVB2 (31V3), CVB3 (20V3), CVB4 (136V3), and CVB5 (03V3) (<1:4). This indicated that there were no cross-neutralizing antibodies between different EV serotypes. However, interestingly, although the CVB1 prototype strain Conn-5 belonged to CVB1 serotype together with our CVB1 isolates, the anti-CVB1 serum could not neutralize the CVB1 prototype strain Conn-5 in the study.

### 3.9. Maternal antibody protection

**Figure 8:**
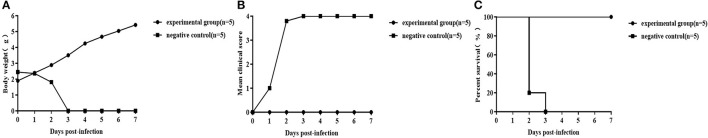
Protective effect of maternal CVB1 antibodies. In total, 8-week-old adult females were immunized twice with 20 μg of the inactivated CVB1 vaccine. After delivery, the 3-day-old mice were inoculated with 10^6^ CCID50 CVB1 strain KM7-X29 P10. The body weight **(A)**, mean clinical score **(B)**, and percent survival **(C)** in each group of neonatal mice were measured. Data were analyzed using the *t*-test and log-rank test.

The efficacy of the inactivated CVB1 vaccine was assessed by the active immunization of 8-week-old female BALB/c mice, followed by challenging their offspring (3-day-old mice) i.c. with KM7-X29 at a dose of 10^6^ CCID50/mouse. It was found that the KM7-X29 (CVB1) group had a normal weight and no significant symptoms during the observation period. Additionally, the survival rate of the group was 100%. However, the mice in the control groups developed clinical symptoms after the KM7-X29 challenge, and all of them died at 3 dpi (Figure 8). These results indicated that the new inactivated CVB1 vaccine could induce humoral immune responses in female mice, and the maternal antibody could provide 100% protection against homologous CVB1 challenge.

## 4. Discussion

An inactivated CVB1 vaccine was previously developed to prevent T1DM associated with CVB1 infection in BALB/c and non-obese diabetic mice (Larsson et al., 2015; Stone et al., 2018). In mice and rhesus macaques, an inactivated hexavalent CVB vaccine could strongly induce the production of NAbs against all six CVB serotypes (Stone et al., 2020). However, the CVB1 strain (Nm/EU147493) used for the vaccine was isolated in 2004 in Finland (Laitinen et al., 2014; Larsson et al., 2015) and belonged to the same cluster (genotype C1) as the CVB1 prototype strain Conn-5. The previous study also found differences in cross-neutralization between the 10 EV-A71 sub-genotypes tested; a C4 genotype EV-A71-based vaccine elicited a powerful cross-neutralization response against other EV-A71 C4 genotypes, and the cross-neutralization of genotype A (EV-A71 prototype strain) was the least efficient. As EV-A71, which included one serotype, five genotypes, and ten sub-genotypes (McMinn, 2012; Ndiaye et al., 2022), CVB1 has one serotype and eight genotypes, although the GII genotype is the most common among them in China. In addition, we found that the antiserum against our inactivated CVB1 vaccine could not efficiently neutralize the CVB1 prototype strain Conn-5, which requires further research. Thus, a new inactivated CVB1 vaccine should be developed to prevent CVB1 infection in China effectively.

Due to the error-prone replication of EVs, they form highly polymorphic populations in their hosts. Since the prototype Conn-5 strain was isolated in 1948, CVB1 isolates have evolved into eight clusters based on the analysis of the whole VP1 gene, so CVB1, like other EVs, is genetically diverse. The Chinese CVB1 strains are divided into clusters GII and GIII; the 20 CVB1 isolates in the present study belong to cluster GII, the prevalent genotype in China. In addition, the average differences in nucleotide and amino acid sequences of the whole VP1 gene between the 20 CVB1 isolates and other Chinese strains available in GenBank were 5.75% (2.20–7.10%) and 2.35% (0.40–4.30%), respectively. The average differences in VP1 nucleotide and amino acid sequences between Chinese strains and the prototype Conn-5 strain or isolates from other countries were substantial (20.80 and 6.50% or 18.25 and 4.55%, respectively). It will provide crucial guidance for screening a CVB1 vaccine candidate by studying the characteristics of different CVB1 clinical isolates in China.

A suitable cell culture system and a highly pathogenic virus strain are essential for the development of inactivated vaccines. Vero cells and KMB17 cells were commonly used for human vaccines such as EV71, Poliovirus, and Hepatitis A virus vaccinations. Tumor-derived cells, such as RD cells, cannot be used for human vaccine production. In our study, nine CVB1 strains isolated from RD cells were removed. In addition, two KMB17 cell-adapted and three Vero cell-adapted strains were also removed because their infectious titers were less than 10^6.0^ CCID50/ml. Numerous inactivated vaccines are produced in Vero cells (Reed and Cardosa, 2016; Green et al., 2021), an extremely safe cellular substrate for vaccine production. By the analysis of the virulence of six CVB1 Vero cell-adapted strains in BALB/c mice, the KM55 strain was the most virulent in BALB/c mice, but the infectious titer was 10^6.25^CCID50/ml. In addition, the inactivated polio vaccines of attenuated strains have proven to be safe and effective and have been successfully used in China (Shimizu, 2016). Considering the cost of vaccine production, the KM7 Vero cell-adapted strain, with the highest virus titer (10^6.875^ CCID50/ml), medium virulent, and the most typical CPE, was screened as a vaccine candidate strain. Then, after three rounds of plaque purification, a clone strain (KM7-X29) was selected because the complete genome sequence of the clone strain is identical to the original strain KM7 by comparing the VP1 gene and complete genome sequences of the 30 clone strains. After 14 serial passages of KM7-X29, the infection titers of KM7-X29 P5, P10, and P15 were all maintained above 10^7.0^ CCID50/ml with the exception of P1(10^5.75^ CCID50/ml), and the complete genome sequences of P10 and P15 were consistent, which revealed that the KM7-X29 strain showed qualified adaptability to Vero cells and was genetically stable after P10. Thus, the KM7-X29 strain was selected as a candidate vaccine strain for further study.

Animal models are crucial for vaccine development and pathogenesis studies. At present, most mouse models are primarily used to evaluate the protective effects of CVB1 vaccines against virus-induced T1DM. In previous research, the RD cell-adapted strain 204 showed strong tropism to the pancreas, but no damage to the heart, brain, liver, limb muscle, and kidney was observed, and in moribund mice, a positive signal was only observed in the heart and spinal cord (Ndiaye et al., 2022). In the present study, histopathology and IHC analyses and tissue viral RNA detection indicated that the KM7-X29 strain had stronger tropism to the pancreas, brain, and liver. Moderate pathological damage was also observed in the heart, limb muscle, and kidney. Previous studies showed that pancreatitis and T1DM were associated with CVB1 infection (Sioofy-Khojine et al., 2018; Centers for Disease Control and Prevention (CDC), 2008) and that CVB1 showed strong tropism to the pancreas (Stone et al., 2018, 2020; Yin et al., 2021). Meningitis, myocarditis, and hepatitis caused by CVB1 infection have been reported in newborns (Wang et al., 1998; Kim et al., 2013). To sum it up, these findings provided further support for the association between CVB1 infection and meningitis, myocarditis, hepatitis, pancreatitis, and T1DM. In addition, previous research also found that different CVB4 isolates could induce different diseases (Benkoova et al., 2021), and CVB has a high frequency of mutations that may result in changes in pathogenicity (Beck et al., 1995); the high mutation frequency of the CVB1 strain might lead to hepatitis (Wang et al., 1998). In addition, the KM7-X29 P10 strain was as pathogenic as its original strain, KM7, in mice (data not shown).

Vaccines must be safe and effective. To determine the immunogenicity of the new inactivated CVB1 vaccine prepared using the KM7-X29 P10 strain, a cross-neutralization test was performed using heterologous CVB1 strains. The results showed that antisera from mice immunized with the inactivated CVB1 vaccine could neutralize all heterologous CVB1 strains. Furthermore, the inactivated CVB1 vaccine could induce the production of protective antibodies in adult female mice, which were passed on to offspring to protect them against lethal doses of CVB1. Moreover, no histopathological change in the pancreas, brain, liver, heart, and limb muscle was observed, although these tissues from the control groups exhibited noticeable pathological changes (data not shown). In addition, no vaccine-related effects were observed. Thus, it is indicated that the new inactivated CVB1 vaccine could protect against meningitis, myocarditis, hepatitis, and pancreatitis, and even T1DM caused by CVB1 infection.

The VP1 protein is the major antigen for EV neutralization. Though amino acid substitutions were observed in the VP1 protein of KM7-X29 offspring and other heterologous CVB1 strains, the inactivated CVB1 vaccine prepared from KM7-X29 P10 could protect against other heterologous CVB1 strains *in vitro*. The whole-genome nucleotide and amino acid sequence homologies of viruses of the P1, P5, P10, and P15 generations were 99.97–100% and 99.90–100%, respectively, which demonstrated that the mutations did not affect their immunogenicity.

Another approach, virus-like particles (VLPs), have been used to develop CVB1 vaccines, which could induce neutralizing antibody responses (Hankaniemi et al., 2019b). VLPs resemble natural viruses in shape and size. However, they lack nucleic acids, are consequently not infectious, and do not revert to a virulent form as the attenuated vaccine. VLPs have good immunogenicity and stimulate immune responses of B and T cells. A promising vaccine candidate is expected to elicit good cellular immunity without side effects. Thus, VLPs may also be another form of CVB1 vaccine development.

In conclusion, a new CVB1 inactivated vaccine candidate strain KM7-X29 was successfully screened, with good pathogenicity, safety, immunogenicity, and genetic stability. Vero cells are the ideal cellular substrate for the inactivated CVB1 vaccine. In addition, it was first reported that CVB1 showed strong tropism for the pancreas, brain, and liver in BALB/c mice.

## Data availability statement

The datasets presented in this study can be found in online repositories. The names of the repository/repositories and accession number(s) can be found in the article/Supplementary material.

## Ethics statement

The animal study was reviewed and approved by Animal Ethics Committee of the Institute of Medical Biology, Chinese Academy of Medical Sciences (DWLL202108001).

## Author contributions

MZ, DX, and YL wrote the manuscript. SM, CF, and WG were involved in the correction and editing of the draft. XW, LX, and NG were involved in the clinical management of patients and data. All authors have read and approved the final manuscript.
