# Supplementary material for: Screening of a new candidate coxsackievirus B1 vaccine strain based on its biological characteristics
Source: Front Microbiol. 2023 Jul 12;14:1172349. doi: 10.3389/fmicb.2023.1172349 (PMC10369069; doi:10.3389/fmicb.2023.1172349)
Supplement: Supplementary file 1 [file Data_Sheet_1.pdf]

Supplementary Material

Evaluation and development of an inactivated coxsackievirus B1 vaccine in mouse

Ming Zhang†[1,2], Danhan Xu†[1,2], Yuhan Liu†[1,2], Xiaohui Wang[1,2], Lilan Xu[1,2], Na Gao[1,2], Changzeng Feng [1,2], Wei Guo[1,2], Shaohui Ma[1,2]*

***Correspondence:** corresponding author Email: shaohuima70@hotmail.com

Table S1. Amplification and sequencing primers of whole genome sequence

primers	sequence（5'→3'）	site
EV1F	TTAAAACAGCCTGTGGGTTG	1-20
B11r	CCTGAGCTCCCATTTCG	751-735
B11f	CAGTGACAAACAGAGCTA	636-653
B12f	CCAGGTAAGTTCACTGAA	886-903
B12R	TTAGTGGGCCGTGAACTAAT	2519-2500
B13f1	CACTCACGGTCCGAATCG	2617-2634
B13f	AACCCAAGCACGTGAAGGCG	3161-3180
B14f	GGCTGGAGGACGATGCAAT	3707-3726
B14f1	CTGTTCTCCAATGTGCAGTAC	4255-4275
B15f	AGATGTTYAGAGARTACAAC	4955-4974
B16f	CGCATGTTGATGTACAAC	5749-5766
EV8R	CACCGAATGCGGAGAATTTA	
B-OS	GGYTAYATNCANTGYTGGTAYCARAC	2296-2321
B-OSA	GGTGCTCACTAGGAGGTCYCTRTTRTARTCYTCCCR	3414-3379
B1-VP1-F	TAATACGACTCACTATAGGGCTCCGT GCGGATGCTGAAGG	2394-2413
B1-VP1-R	CATATATTGCTCCGGACTGCTG	3316-3295
CVB1-qPF	GACTGGGCATACCTCCCAAGT	2553-2573
CVB1-qPR	ATTCGGACCGTGAATGGTAGTTT	2632-2610
B1-DB-probe	6-FAM－CCTAGYGACACCA TGCAAACAAGACAYG－BHQ1	2578-2605
M13F	TGTAAAACGACGGCCAGT	
M13R	CAGGAAACAGCTATGACC	

Note: Position reference sequence KM7-X29 P1. F/f is the upstream primer, R/r is the downstream primer; B-OS is the upstream primer, B-OAS is the downstream primer; the underline indicates the T7 promoter sequence; B1-DB-probe is the probe.

Table S2. Source and isolated cell lines of 20 CVB1 samples

strain	year of separation	clinical sample	cell line
R461	2010	faeces	RD
R42	2014	faeces	RD
R43	2014	faeces	RD
K148	2014	faeces	KMB-17
V1444	2014	faeces	Vero
R157	2015	faeces	RD
K1512	2015	faeces	KMB-17
R1516	2015	faeces	RD
R1539	2015	faeces	RD
R1552	2015	faeces	RD
R1570	2015	faeces	RD
V1573	2015	faeces	Vero
V1578	2015	faeces	Vero
R1587	2015	faeces	RD
KM7	2019	faeces	Vero
KM46	2019	faeces	Vero
KM53	2019	faeces	Vero
KM55	2019	faeces	Vero
KM62	2019	faeces	Vero
KM64	2019	faeces	Vero

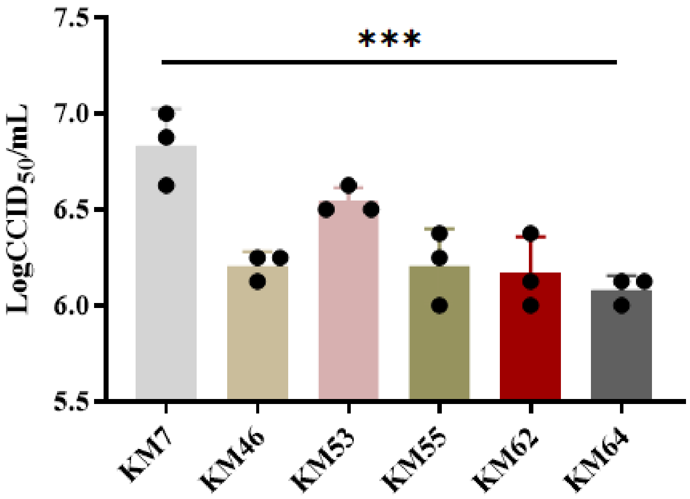

Figure S1. Infectious titers of the different CVB1 isolates. Data were statistically analyzed using one-way ANOVA, *** indicates $p < 0.001$.

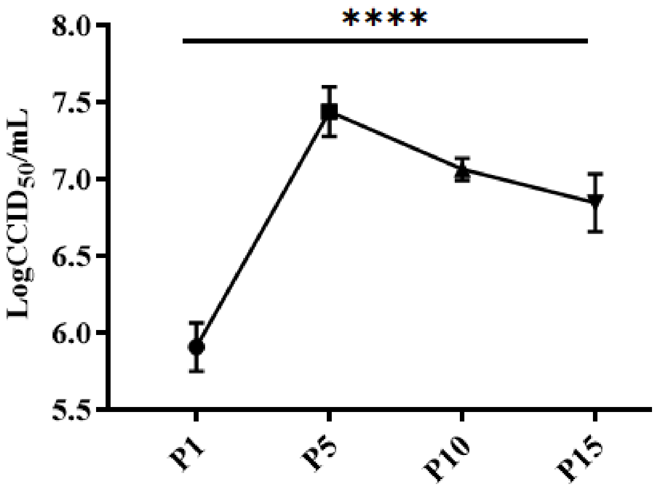

Figure S2. Infectivity titers of KM7-X29 strain P1, P5, P10 and P15 generation. Data were statistically analyzed using one-way ANOVA, **** indicates $p < 0.0001$.
